# Prospective Comparison of 18F-Choline Positron Emission Tomography/Computed Tomography (PET/CT) and 18F-Fluorodeoxyglucose (FDG) PET/CT in the Initial Workup of Multiple Myeloma: Study Protocol of a Prospective Imaging Trial

**DOI:** 10.2196/17850

**Published:** 2020-09-10

**Authors:** Charles Mesguich, Cyrille Hulin, Valerie Latrabe, Julien Asselineau, Laurence Bordenave, Paul Perez, Elif Hindie, Gerald Marit

**Affiliations:** 1 Nuclear Medicine Department Centre Hospitalier Universitaire de Bordeaux Pessac France; 2 INSERM U1035 Université de Bordeaux Bordeaux France; 3 Hematology Department Centre Hospitalier Universitaire de Bordeaux Pessac France; 4 Radiology Department Centre Hospitalier Universitaire de Bordeaux Pessac France; 5 Clinical Epidemiology Department Centre Hospitalier Universitaire de Bordeaux Pessac France

**Keywords:** multiple myeloma, PET/CT, MRI, 18-FDG, 18F-choline, cancer, medical imaging, skeletal system

## Abstract

**Background:**

The International Myeloma Working Group recommends the use of 18-fluorodeoxyglucose (FDG) positron emission tomography/computed tomography (PET/CT) for treatment response evaluation, as it is superior to magnetic resonance imaging (MRI). However, at initial staging, the sensitivity of FDG-PET remains inferior to that of MRI. Therefore, there is a need for an imaging technique that could have a sensitivity equal to that of MRI at diagnosis and could serve to evaluate therapy. 18F-choline has shown increased sensitivity when compared with 18-FDG, with about 75% more lesions detected in patients with relapsed or progressive multiple myeloma (MM).

**Objective:**

Our primary objective is to prospectively compare the detection rate of bone lesions by 18F-choline PET/CT (FCH-PET) and FDG-PET in newly diagnosed MM. Our secondary objectives are to assess the accuracy of both PET modalities for the detection of bone lesions and the diagnosis of diffuse disease, to assess the detection rate of extramedullary lesions.

**Methods:**

We will prospectively include 30 patients in a paired comparative accuracy study. Patients with de novo MM will undergo FCH-PET, FDG-PET, and whole-body MRI (WB-MRI) within a 3-week period. WB-MRI will be composed of conventional sequences on the spine and pelvis and of whole-body diffusion axial sequences. The following 6 skeletal areas will be defined: skull, sternum/costal grid, spine, pelvis, superior limbs, and inferior limbs. The number of focal lesions, their respective localization, and intensity of uptake will be retrieved for each skeletal area. Readings will be performed blinded from other imaging techniques. The reference standard will be WB-MRI. Focal lesions present on PET/CT but not on WB-MRI will require a decision made with a consensus of experts based on clinical and imaging data. The number of bone lesions and number of extramedullary lesions will be compared using the Wilcoxon test. The accuracy of FCH-PET and FDG-PET will be compared using the McNemar test.

**Results:**

The study started in September 2019, and enrollment is ongoing. As of June 2020, 8 participants have been included. Data collection is expected to be completed in June 2021, and the results are expected to be available in December 2021.

**Conclusions:**

This study will assess if FCH-PET is superior to FDG-PET for the evaluation of MM tumor burden. This will pave the way for future prospective evaluations of the prognostic value of 18-FCH for treatment response evaluation in MM patients. Additionally, this work may provide new perspectives for better assessment of the risk of smoldering MM progressing to MM.

**Trial Registration:**

ClinicalTrials.gov NCT03891914; https://clinicaltrials.gov/ct2/show/NCT03891914

**International Registered Report Identifier (IRRID):**

DERR1-10.2196/17850

## Introduction

Multiple myeloma (MM) is the second most frequently occurring hematological malignancy, with an incidence rate in Europe of 7.4/100,000 men per year and 3.8/100,000 women per year [1,2]. The median age at diagnosis is 69 years [2,3].

MM is defined by the clonal proliferation of more than 10% of malignant plasma cells in the bone marrow associated with signs of myeloma-related organ dysfunction including anemia, hypercalcemia, bone lesions, and renal impairment.

During the past decade, the median overall survival period has almost doubled, from a median of 3 years to 6 years, mainly due to the expansion of the therapeutic arsenal with the use of proteasome inhibitors and immunomodulatory drugs [4]. While the proportion of patients reaching a complete response has increased, the majority of patients eventually relapse. The survival of patients with MM mainly depends on prognostic factors at initial workup. These factors include age, performance status, extramedullary disease, the revised International Staging System stage, cytogenetic abnormalities, and tumor burden [5]. Another important prognostic factor is the depth of the biological response to treatment. This is currently assessed by measuring the level of secreted monoclonal protein in the blood or urine. Complementarily, evaluation of minimal residual disease (MRD) is increasingly performed to finely evaluate patient’s treatment response with the use of multiparameter flow cytometry or next-generation sequencing [6]. However, MRD assessment requires a sample of bone marrow, usually taken from the iliac crests. MRD diagnostic performance can therefore be hampered by the location of residual disease.

Functional imaging such as 18- fluorodeoxyglucose (FDG) positron emission tomography/computed tomography (PET/CT) or magnetic resonance imaging (MRI) can help assess the tumor burden at diagnosis as well as residual disease. Previous studies have shown that 18-FDG PET/CT is superior to MRI for the evaluation of treatment response [7-12]. However, its diagnostic value at initial workup can be challenging for different reasons. First, FDG uptake by myeloma cells can be low because of variations in the glucose metabolism pathway [13]*.* Second, the depiction of skull lesions is generally hampered because of the high physiological FDG uptake of the brain [14]. Third, 18-FDG PET/CT can miss diffuse infiltration [12,15,16]. Therefore, whole-body MRI (WB-MRI) remains more sensitive than 18-FDG PET/CT for the initial diagnosis of MM [13].

Therefore, finding a radiotracer that could have superior diagnostic value at initial workup would allow for better discrimination of the initial tumor burden and hopefully also be useful to better evaluate the treatment response. Recent years have seen the advent of novel metabolic tracers such as 11C-methionine and 11C-labelled or 18F-labelled choline [17-19].

18F-choline, a tracer of phospholipids in the cell membrane, has good availability, as it is used for prostate cancer imaging. In a recent study, 18F-choline showed potential as compared to 18-FDG in MM, with about 75% more lesions detected in patients with MM with suspected relapsing disease [18]. 18F-choline has no physiological uptake in the brain, allowing optimal evaluation of the skull. Also, in this study, the median uptake of 18F-choline was superior to that of 18-FDG [18]. However, the retrospective design and heterogeneous population of this study warrant confirmation from prospective studies. Furthermore, as no reference standard was defined, the diagnostic performance of each modality could not be derived and compared.

Therefore, the current prospective diagnostic accuracy study was designed to compare, in patients with de novo MM, the number of bone lesions detected by 18-FDG PET/CT and 18F-choline PET/CT, with confirmation by a reference standard, in the entire body as well as in predefined skeletal areas (skull, spine, pelvis, ribs-sternum, superior limbs, inferior limbs).

## Methods

**Figure 1:**
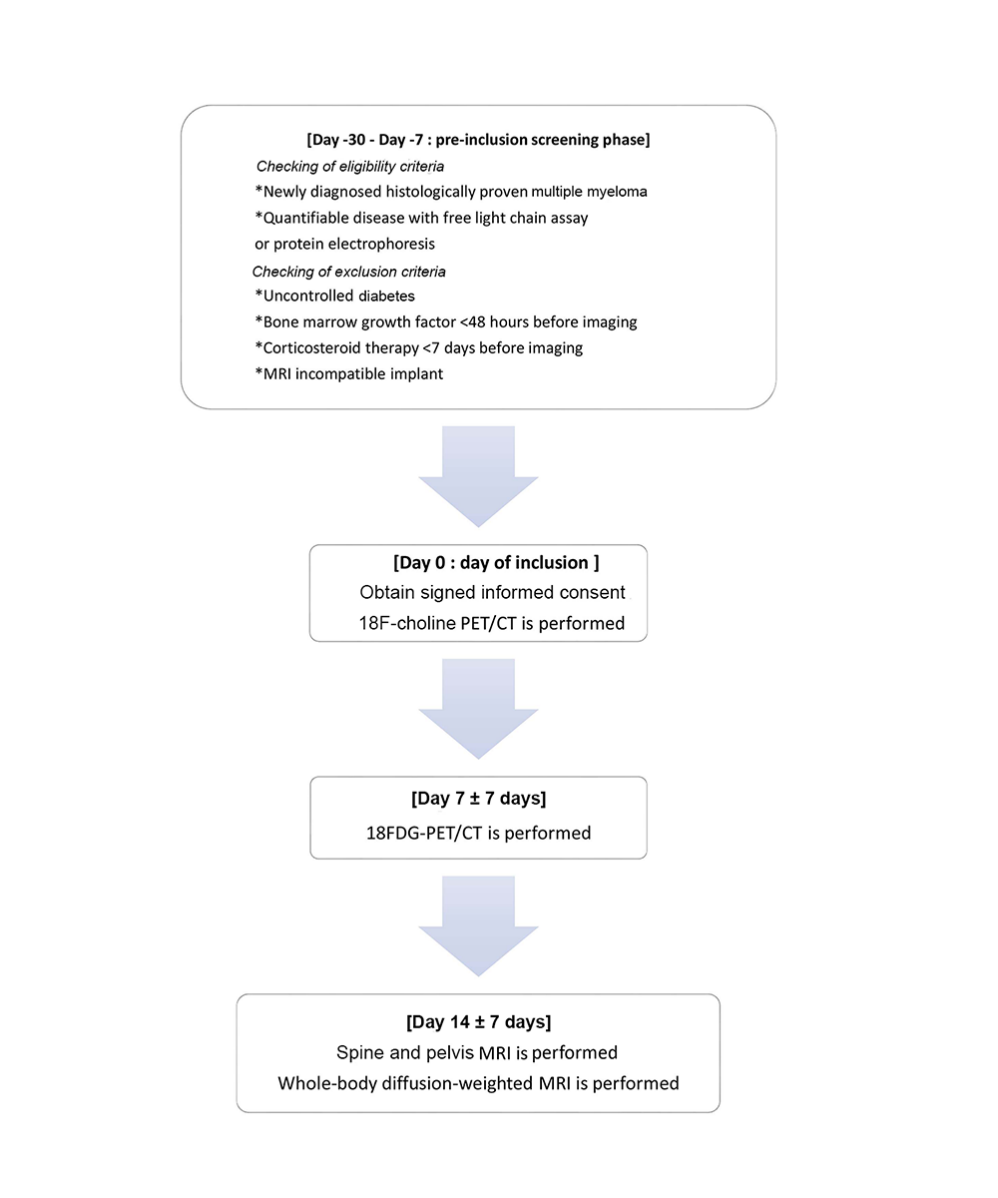
Flowchart of the current prospective diagnostic trial. CT: computed tomography; FDG: fluorodeoxyglucose; MRI: magnetic resonance imaging; PET: positron emission tomography.

The scheme of the current prospective trial is shown in Figure 1, and the trial is described hereafter. Within a 21-day period, patients undergo 3 imaging procedures in the following order: 18F-choline PET/CT, 18-FDG PET/CT, and WB-MRI. The second and third imaging procedures will be performed 7 days ± 7 days from the previous procedure.

### Patient Selection

#### Inclusion Criteria

Patients with a de novo diagnosis of MM as defined by the International Myeloma Working Group [20] will be prospectively and consecutively recruited at the University Hospital of Bordeaux. Patients must have quantifiable disease, either by the monoclonal component in blood or urine or by free light chain assay. Patients must be older than 18 years and be affiliated with the French social security regimen (or other health insurance regimen). Women of childbearing age must provide a negative pregnancy test before undergoing PET/CTs. Finally, protocol approval signed by the patient and the principal investigator must be obtained by the principal investigator before inclusion.

#### Exclusion Criteria

Subjects will be excluded if they had any other cancer history in the previous 5 years or if they already had received myeloma treatment. To obtain optimal imaging assessments, we will exclude patients with uncontrolled diabetes, patients that had received injections of bone marrow stimulating growth factor <72 hours before proceeding to PET/CT or MRI, and patients that had received corticosteroids during the 7 days preceding PET/CT. Also, patients with MRI-incompatible implants will be excluded.

### 18F-Choline PET/CT

#### Imaging Protocol

Patient must fast for 6 hours before the procedure. 18F-choline is injected with an activity of 3 MBq/kg. Whole-body PET/CT acquisition (from vertex of the skull to the knee) is performed 10 minutes after injection.

#### Interpretation Criteria

After anonymization, images will be independently read by two experienced nuclear medicine physicians, blinded from clinical data, biological data, and other imaging data performed during the protocol.

18F-choline has physiological uptake by the liver, spleen, pancreas, salivary glands, and urinary tract. Lymph node uptake will not necessarily be considered pathological, particularly in the mediastinum, as lymphadenitis and granulomatosis are sources of false positive findings. Any focal bone uptake will be considered pathological if the uptake is superior to that of surrounding background activity. The following 6 skeletal areas will be defined: skull, sternum-ribs, spine, pelvis, superior limbs, and inferior limbs. The number of focal lesions (FL), localization, and intensity of uptake (SUVmax) will be described for each skeletal area. Diffuse infiltration of the spine will be defined as diffuse spine uptake that is superior to liver uptake [11,14].

### 18-FDG PET/CT

#### Imaging Protocol

Patients must fast for 6 hours before proceeding to imaging. 18-FDG is injected with an average dose of 3 MBq/kg. PET/CT is performed from the vertex to the knees 60 minutes after injection.

#### Interpretation Criteria

After anonymization, images will be independently read by two experienced nuclear medicine physicians, blinded from clinical data, biological data, and other imaging data performed during the protocol.

18-FDG has physiological uptake by the brain, myocardium, kidneys, urinary tract, liver, and spleen. A focal bone lesion is defined as a focal uptake that is superior to surrounding background uptake. An extramedullary lesion will be considered if there is focal uptake within organ or lymph node structures. Of note, bilateral mediastinal lymph node uptake will not be considered as disease, as this will more likely correspond to granulomatosis lesions like sarcoidosis. The same 6 skeletal areas will also be used to classify focal bone lesions. The number of FL, localization, and SUVmax will be retrieved for each skeletal area. Diffuse infiltration will be defined as diffuse spine uptake that is superior to liver uptake [11,14].

### Whole-Body MRI (WB-MRI)

#### Imaging Protocol

WB-MRI examinations will be performed on a 1.5 Tesla device (Aera, Siemens Healthineers, Erlangen, Germany). First, the following imaging protocol will be applied: coronal T1-weighted turbo spin echo sequences on the pelvis (repetition time [TR] 785 ms; echo time [TE] 10 ms), coronal T2-weighted short-term inversion recovery (STIR) sequences on the pelvis (TR 13850 ms; TE 90 ms), sagittal T1-weighted turbo spin echo sequences on the spine (TR 453 ms; TE 12 ms), and sagittal T2-weighted STIR sequences (TR 7450 ms; TE 62 ms). Second, axial diffusion-weighted (DW) sequences will be acquired from the vertex to knees in 7 to 9 stacks. We will apply *b* values of 50 s/mm^2^ and 800 s/mm^2^. Each stack will be composed of 50 slices of 5-mm thickness (TR 7959 ms; TE 61 ms; inversion time 180 ms). Fused whole-body 3D maximal intensity projection of DW images will be built for analysis. The total length of the MRI protocol will be 45 minutes.

#### Interpretation Criteria

After anonymization, MRI images will be reviewed by two experienced radiologists blinded from PET/CT, clinical, and biological data. An FL is defined on coronal and sagittal sequences as a lesion with a diameter of more than 5 mm, with a low signal intensity on T1-weighted imaging and a high signal intensity on T2-weighted imaging. On DW images, an FL is defined as focal intensity above the bone marrow background signal. Diffuse bone marrow infiltration is defined based on previously published criteria [21]: a vertebral body to vertebral disk signal ratio <1.3 on T1-weighted images, vertebral body to psoas muscle signal ratio >2 on T2-weighted images, and vertebral body to kidney signal ratio >1 on DW images. A mild infiltration is defined as a pathological finding on T2-weighted or DW sequences. A moderate infiltration is defined as a pathological finding on both T2-weighted and DW images; a severe infiltration is defined as a pathological finding on both aforementioned sequences and on T1-weighted images.

### Reference Standard

The reference standard is WB-MRI, which is comprised of conventional MRI sequences combined with WB-MRI diffusion acquisition. Each FL found on 18F-choline or 18-FDG PET/CT and not present on MRI will undergo multidisciplinary consensus between hematologists, nuclear medicine physicians, and radiology physicians. A decision will be reached and will classify an FL as a true-positive or false-positive finding based on patient charts and results from 18-FDG PET/CT, 18F-choline PET/CT, and WB-MRI at baseline but also based on lesion response to chemotherapy during a 12-month follow-up.

### Primary Outcome Measures

#### Number of Whole-Body Bone Lesions on 18F-Choline PET/CT and 18-FDG PET/CT

Every bone lesion will be validated using the reference standard, which is WB-MRI. A bone lesion that is not present on MRI but is present on any of the PET modalities must be validated by an expert multidisciplinary consensus.

#### Number of Bone Lesions Within Defined Skeletal Areas on 18F-Choline PET/CT and 18-FDG PET/CT

The following 6 skeletal areas will be defined: skull, spine, pelvis, sternum and ribs, superior limbs, inferior limbs. The number of bone lesions in each of these skeletal areas is assessed. Each bone lesion must be validated as mentioned in the previous paragraph.

### Secondary Outcome Measures

#### Diagnostic Accuracy of 18F-Choline PET/CT and 18-FDG PET/CT for the Detection of Focal Bone Lesions

Sensitivity, specificity, positive and negative predictive values, and diagnostic likelihood ratios of each test will be calculated based on two different reference tests. The first reference test will be standard MRI sequences (T1-weighted turbo spin echo sequences, T2-STIR). The second reference test will be a reference standard composed of standard MRI sequences plus DW WB-MRI acquisitions.

#### Diagnostic Accuracy of 18F-Choline PET/CT and 18-FDG PET/CT for the Detection of Diffuse Infiltration of the Spine

Sensitivity, specificity, positive predictive value, negative predictive value, and diagnostic likelihood ratios of each test will be calculated based on WB-MRI results.

#### Number of Extramedullary Lesions on 18F-Choline PET/CT and 18-FDG PET/CT

The number of extramedullary lesions detected by each test will be compared.

### Sample Size Calculation and Statistics

The main objective is to compare the number of FL detected on 18F-choline PET/CT and 18-FDG PET/CT, after confirming that each lesion exists on the reference standard. Data from a recent article showed that 18F-choline detects 75% more bone lesions than 18-FDG PET/CT [22]. Intrapatient concordance was 0.85. For each tracer, the standard error and mean of the distribution of the number of lesions wsere equal. We hypothesized that we will observe an average number of 10 bone lesions on 18-FDG PET/CT and that this number will increase by 75% on 18F-choline.

With a type I error of 5% and power of 90%, it will be necessary to include 23 patients. Due to the probability that the number of lesions will not follow a normal distribution, we will use a Wilcoxon signed-rank test, which is less powerful than the Student *t* test. To compensate for this lack of power, we will include 30 patients.

The secondary objectives are to compare the global accuracy of 18F-choline PET/CT and 18-FDG PET/CT as well as the accuracy of these two modalities in each of the 6 skeletal areas defined earlier for the detection of MM bone lesions. Accuracies of each modality will be compared using the McNemar test. Other secondary objectives will include the comparison of the accuracies of 18F-choline PET/CT and 18-FDG PET/CT for detecting diffuse infiltration of the spine using the McNemar test. Comparison of the number of extramedullary lesions detected by 18-FDG PET/CT and 18F-choline PET/CT will be performed using the Wilcoxon signed-rank test.

Interobserver and intraobserver agreements of PET/CT readings will be evaluated using the Cohen kappa coefficient.

### Research Monitoring

#### Scientific Research Council

The Scientific Research Council meets according to the needs of the study and at least once a year. Its mission is to make any important decision at the request of the coordinating investigator regarding the smooth running of research and compliance with the protocol. It enquires about the research progress, possible issues, and available results at the Center for Methodology and Data Management and the research coordinating center of the state. The council makes decisions about any relevant modification of the protocol necessary for the continuation of the research; in particular, the council make decisions about strategies to facilitate recruitment in research, discussion of the results, and the publication strategy of these results. The Scientific Research Council can propose to prolong or interrupt the research if the rate of inclusion is too slow, if there are too many people lost to follow-up, if there are too many major violations of the protocol, or for medical or administrative reasons.

#### Independent Data Monitoring Committee

The only investigation procedure for this research is 18F-choline PET-CT. 18F-choline has had marketing authorization in France since 2010 and is used in clinical routine for the search for metastases in prostatic adenocarcinoma and for the extension assessment of well-differentiated hepatocellular carcinomas. There are no adverse effects reported to date with this radiotracer. Given these elements, the study does not pose any additional risk for the study participants. In conclusion, an independent monitoring committee is not necessary.

### Data Collection and Management

#### Instructions for Data Collection

All information and data required by the protocol will be recorded in paper notebooks, and an explanation should be provided for each piece of missing data. The data must be collected as soon as they are obtained and clearly and legibly transcribed in these notebooks.

#### Data Management

Data are double entered. The first entry is made using EpiData (EpiData Association, Odense, Denmark), and validation is performed via a different operator using DBS software (DBS Software and Services, Clearwater, MI). In case of needed transfer, data will be transferred to the Methodology and Data Management Center via a secure FTP channel.

Software used are ACCESS (SAS Institute Inc, Cary, NC) and SAS (SAS Institute Inc, Cary, NC).

The study data remain stored on the server of the director of information systems of the Bordeaux University Hospital, in accordance with the current regulations. A physical copy is kept by the promoter in accordance with the current regulations.

### Deviations to the Protocol

#### Participants Lost to Follow-Up

A participant is considered lost to follow-up when he or she stops the planned 12-month follow-up under the protocol without a reason known to the investigator, so that data collection cannot proceed as planned. The absence of news for more than 1 month defines lost to follow-up.

Data for participants lost to follow-up will be actively researched by the investigator.

#### Participants Incorrectly Included

A participant is considered to be wrongly included when he or she was actually included in the search even though he or she did not meet all the eligibility criteria. Participants wrongly included should be discussed with the Scientific Research Council. They must continue to be monitored as per the protocol until a decision is made by the Scientific Research Council.

### Management of Adverse Events and Other Unintended Effects

The investigator is responsible for the collection of adverse events that occur between the date of written consent and the end of the participant's participation. The investigator must notify the security and vigilance unit by fax or mail, without delay as of the day when he knows of any serious adverse event. The security and vigilance unit shall immediately declare new facts that have arisen during the search to the French National Agency for the Security of Drugs and Health Products (ANSM) and the Committee for the Protection of Persons (CPP).

### Annual Security Report

On the anniversary date of the research authorization, the security and vigilance unit will create a security report comprising the list of serious adverse events likely to be related to the experimental procedures including expected and unexpected serious events that occurred during the trial during the period covered by the report, which will include a concise and critical analysis of the safety of the participants who are suitable for research. This report is sent to the ANSM and the CPP within 60 days of the anniversary date of the authorization of research.

### Ethical and Regulatory Considerations

The sponsor and investigator undertake to ensure that this research is conducted in accordance with the law relating to research involving the human person (n° 2012-300 of March 5, 2012) as well as in agreement with Good Clinical Practice (International Conference on Harmonization version 4 dated November 9, 2016 and decision dated November 24, 2006) and the Declaration of Helsinki [23].

This research received the favorable opinion of the CPP Sud Méditerrannée I and the authorization of the ANSM. The University Hospital of Bordeaux, sponsor of this research, has signed a liability insurance contract with Gerling-Biomedicinsure in accordance with the provisions of the Public Health law. The data recorded during this research are the subject of computerized processing at the methodological support unit for clinical and epidemiological research in compliance with law n ° 78-17 (dated January 6, 1978) relating to computers, files, and freedoms amended by law 2004-801 (dated August 6, 2004).

### Amendments to the Protocol

Any substantial modification must receive, prior to its implementation, a favorable opinion from the CPP and an authorization from the ANSM. Non-substantial changes (ie, those that have no significant impact on any aspect research) are communicated to the CPP for information purposes. All changes are validated by the sponsor and by all research stakeholders concerned before submission to the CPP and ANSM.

## Results

The study started in September 2019, and enrollment is ongoing. As of June 2020, 8 participants have been included. Data collection is expected to be completed in June 2021, and the results are expected to be available December 2021.

## Discussion

### Overview

A recent study showed that the rate of false-negative results for 18-FDG PET/CT for the detection of bone disease in MM is about 10%, which could be attributed to a lower expression of hexokinase 2 [13]. A more sensitive tracer is therefore needed.

The role of 18F-choline in the management of relapsed prostate cancer is well-established, and it also has a role in the characterization of hepatocellular carcinoma. However, it remains poorly explored in the field of hematological diseases. 18F-choline is incorporated into the membrane of cells in division and could reflect the higher rate of neoplastic plasma cells.

A recent retrospective study has shown that 18F-choline detects 75% more focal bone uptakes than 18-FDG PET/CT [18]. However, this study was performed on a heterogeneous previously treated population with suspected progressive disease or relapse. It is known that 18-FDG metabolism in myeloma cells can change after first-line treatment and that FDG-negative myeloma cases at diagnosis can turn FDG-positive when they relapse [13].

This prospective comparative study of diagnostic accuracies will evaluate if 18F-choline PET/CT is superior to 18-FDG PET/CT in a homogenous population with de novo MM. The expected role (according to STARD 2015 definitions) of 18F-choline PET/CT is the replacement of 18-FDG PET/CT. The study has been designed to comply with QUADAS 2 guidelines. We will carefully exclude patients that are already being treated with corticosteroids because of the increased risk of false-negative findings on 18-FDG PET/CT and patients that had a recent injection of bone marrow stimulation factors because of the risk of a false-positive assessment of bone marrow on PET/CT and MRI [14]. Also, 18-FDG PET/CT, 18F-choline, and WB-MRI will be performed within a short time period to avoid misinterpretation due to the apparition of new bone lesions during the protocol.

By analyzing the number of bone lesions per skeletal area, we expect to find that 18F-choline is superior to 18-FDG PET/CT for the detection of bone lesions, especially in the skull, as 18F-choline has no brain uptake. A previous study showed that the median uptake of 18F-choline is higher than that of 18-FDG [18]. Hence, we expect that 18F-choline PET/CT will be more sensitive than 18-FDG PET/CT for the detection of focal bone lesions as well as for the detection of diffuse infiltration.

Several studies have demonstrated that 18-FDG PET/CT remains the most performant imaging procedure for the assessment of MM response to chemotherapy [7-9,11]. However, treatment response assessment would benefit from a baseline evaluation that would depict all existing lesions. Hence, the current study will pave the way for future prospective studies that will aim at evaluating 18F-choline as a tool to evaluate treatment response in patients with MM, in comparison with 18-FDG PET/CT. Therefore, there is hope that we can find a “one-stop-shop” imaging procedure that would perform equally to MRI at diagnosis and with a prognostic value equal or superior to 18-FDG PET/CT.

Smoldering multiple myeloma (SMM) is defined as clonal plasma cell proliferation with 10-60% of plasma cells in the bone marrow, without organ damage. No treatment is required until it reaches the MM stage. The risk of SMM progressing to MM is ~10% each year during the first 5 years [20]. In patients without MM bone lesions on CT, it has been demonstrated that SMM presenting with hypermetabolic foci within the bone had higher chances of progression to MM than without focal uptake [24]. Hence, 18-FDG PET/CT could help stratify the risk of SMM progression to MM. The present prospective study will provide perspectives for the evaluation of SMM with 18F-choline, as a more sensitive tracer would allow even better characterization of the prognosis of patients with SMM.

### Conclusions

This study will assess if 18F-choline PET/CT is superior to 18-FDG PET/CT for the evaluation of MM tumor burden. This will pave the way for future prospective evaluations of the prognostic value of 18F-choline PET/CT to evaluate the treatment response in patients with MM. Additionally, this work may provide new perspectives for better assessment of the risk of SMM progressing to MM.

## Appendix

Multimedia Appendix 1 External reviewing 1.

Multimedia Appendix 2 External reviewing 2.

## Abbreviations

ANSM: French National Agency for the Security of Drugs and Health Products
CPP: Committee for the Protection of Persons
CT: computed tomography
DW: diffusion-weighted
FDG: fluorodeoxyglucose
MM: multiple myeloma
MRD: minimal residual disease
MRI: magnetic resonance imaging
PET: positron emission tomography
SMM: smoldering multiple myeloma
STIR: short-term inversion recovery
TE: echo time
TR: repetition time
WB-MRI: whole-body MRI
