# Supplementary material for: Prospective Comparison of 18F-Choline Positron Emission Tomography/Computed Tomography (PET/CT) and 18F-Fluorodeoxyglucose (FDG) PET/CT in the Initial Workup of Multiple Myeloma: Study Protocol of a Prospective Imaging Trial
Source: JMIR Res Protoc. 2020 Sep 10;9(9):e17850. doi: 10.2196/17850 (PMC7516691; doi:10.2196/17850)
Supplement: Multimedia Appendix 1 [file resprot_v9i9e17850_app1.pdf]

APPEL D'OFFRES INTERNE 2017

Fiche d'évaluation du projet complet

PORTEUR DU PROJET : Charles Mesguich

TITRE DU PROJET : COMPARAISON PROSPECTIVE, LORS DU BILAN D'EXTENSION INITIAL DE MYELOME MULTIPLE, DE LA TEP-TDM A LA 18F-FLUOROCHOLINE ET DE LA TEP-TDM AU 18F-FDG

1. Originalité

☐ **Aucune** ☐ **Partielle** X☐ **Totale**

Commentaire argumenté (détailler les points forts et les points faibles)
Ce protocole serait la premiere étude prospective évaluant la TEP à la FCHOLINE dans le MM au diagnostic, avec pour gold standard l'IRM. Situation dans laquelle la TEP au FDG peut manquer de sensibilité.

2. Hypothèse, Méthodologie

Hypothèse scientifique clairement mentionnée	x☐ Oui	☐ Non
Objectif principal clair, en cohérence avec l'hypothèse principale	x☐ Oui	☐ Non
Critère d'évaluation principal : fiable, pertinent	x☐ Oui	☐ Non
Schéma et méthodes pertinents pour l'hypothèse et l'objectif principal	x☐ Oui	☐ Non
Critères d'inclusion et non inclusion : clairs, adaptés	x☐ Oui	☐ Non
Calcul du nombre de sujets nécessaire : clair, adapté	x☐ Oui	☐ Non

Commentaire argumenté (détailler les points forts et les points faibles)
Objectifs et criteres de jugement clairement énoncés.
Discuter une acquisition TEP plus précoce apres l' injection de FCHOLINE (10 -20 minutes) comme effectué dans la reference retrospective de l'équipe de Tenon.

3. Faisabilité

Potentiel de recrutement dans le temps imparti par l'AOI (2 ans)	x ☐ Oui	☐ Non
Maîtrise des circuits (produits, patients, prélèvements…)	x ☐ Oui	☐ Non

Commentaire argumenté (détailler les points forts et les points faibles)
Au vu du nombre de cas de MM de novo dans le centre (180/an), le recrutement de 30 patients /18 mois est atteignable.
La réalisation de TEP au FDG et a la fluorocholine est de pratique courante, sans difficulté particuliere.

4. Qualité rédactionnelle

☐ **Faible** ☐ **Bonne** x ☐ **Très bonne**

Commentaire argumenté (détailler les points forts et les points faibles)

5. Retombées attendues

En termes de connaissances scientifiques	x☐ Oui	☐ Non
Conséquences en santé publique et/ou pour les patients	x☐ Oui	☐ Non

Commentaire argumenté (détailler les points forts et les points faibles)

6. Aspects budgétaires

Adéquation des crédits demandés*	☐ Oui	☐ Non

Commentaire argumenté (détailler les points forts et les points faibles)
Les surcouts de l'examen a la flurorcholine sont pris en compte.

Conclusion
Tres bon projet d'imagerie fonctionelle qui pourra déboucher sur des travaux de recherche d'évaluation de la réponse au traitement.

Projet de phase pilote permettant d'envisager la soumission à un appel à projets national à venir x☐ Oui ☐ Non

Liens d’intérêt et confidentialité

x☐ Je m’engage à respecter la plus stricte confidentialité concernant ce projet

x☐ Je déclare avoir examiné ce projet en l’absence de lien d’intérêt avec le projet, le porteur de son projet et son équipe.

☐ Je déclare les liens d’intérêts potentiels suivants :

Type de lien	Dates	Commentaire

* Les coûts de Promotion ne sont pas éligibles à l’Appel d’offres Interne (soutien technico-réglementaire, monitoring, vigilance, méthodologie).
