# Supplementary material for: Prospective Comparison of 18F-Choline Positron Emission Tomography/Computed Tomography (PET/CT) and 18F-Fluorodeoxyglucose (FDG) PET/CT in the Initial Workup of Multiple Myeloma: Study Protocol of a Prospective Imaging Trial
Source: JMIR Res Protoc. 2020 Sep 10;9(9):e17850. doi: 10.2196/17850 (PMC7516691; doi:10.2196/17850)
Supplement: Multimedia Appendix 2 [file resprot_v9i9e17850_app2.pdf]

# APPEL D'OFFRES INTERNE 2017

Fiche d'évaluation du projet complet

**PORTEUR DU PROJET** : Charles Mesguich

**TITRE DU PROJET** : COMPARAISON PROSPECTIVE, LORS DU BILAN D'EXTENSION INITIAL DE MYELOME MULTIPLE, DE LA TEP-TDM A LA 18F-FLUOROCHOLINE ET DE LA TEP-TDM AU 18F-FDG

**1. Originalité**

☐ **Aucune** ☐ **Partielle** ☒ **Totale**

Commentaire argumenté (détailler les points forts et les points faibles)

**2. Hypothèse, Méthodologie**

| | | |
|---|---|---|
| Hypothèse scientifique clairement mentionnée | ☒ Oui | ☐ Non |
| Objectif principal clair, en cohérence avec l'hypothèse principale | ☒ Oui | ☐ Non |
| Critère d'évaluation principal : fiable, pertinent | ☒ Oui | ☐ Non |
| Schéma et méthodes pertinents pour l'hypothèse et l'objectif principal | ☒ Oui | ☐ Non |
| Critères d'inclusion et non inclusion : clairs, adaptés | ☒ Oui | ☐ Non |
| Calcul du nombre de sujets nécessaire : clair, adapté | ☒ Oui | ☐ Non |

Commentaire argumenté (détailler les points forts et les points faibles)
**Sujet original et pertnent d'un point de vue clinique, très bonne methodologie, réaliste pour un AOI**

**3. Faisabilité**

| | | |
|---|---|---|
| Potentiel de recrutement dans le temps imparti par l'AOI (2 ans) | ☒ Oui | ☐ Non |
| Maîtrise des circuits (produits, patients, prélèvements…) | ☒ Oui | ☐ Non |

Commentaire argumenté (détailler les points forts et les points faibles)
Recrutement tout à fait réaliste

**4. Qualité rédactionnelle**

☐ **Faible** ☐ **Bonne** ☒ **Très bonne**

Commentaire argumenté (détailler les points forts et les points faibles)
Très bonne qualité rédactionnelle

**5. Retombées attendues**

| | | |
|---|---|---|
| En termes de connaissances scientifiques | ☒ Oui | ☐ Non |
| Conséquences en santé publique et/ou pour les patients | ☒ Oui | ☐ Non |

Commentaire argumenté (détailler les points forts et les points faibles)

**6. Aspects budgétaires**

| | | |
|---|---|---|
| Adéquation des crédits demandés* | ☒ Oui | ☐ Non |

Commentaire argumenté (détailler les points forts et les points faibles)
Budget calculé de façon réaliste avec soutien industriel

Conclusion
Excellent projet,claireemnt présenté, réaliste avec retombées cliniques dans la prise en charge du myelome

Projet de phase pilote permettant d'envisager la soumission à un appel à projets national à venir ☒ Oui ☐ Non

## Liens d’intérêt et confidentialité

☒ Je m’engage à respecter la plus stricte confidentialité concernant ce projet

☒ Je déclare avoir examiné ce projet en l’absence de lien d’intérêt avec le projet, le porteur de son projet et son équipe.

☐ Je déclare les liens d’intérêts potentiels suivants :

| Type de lien | Dates | Commentaire |
|---|---|---|
| | | |
| | | |
| | | |
| | | |

* Les coûts de Promotion ne sont pas éligibles à l’Appel d’offres Interne (soutien technico-réglementaire, monitoring, vigilance, méthodologie).
